# Ontogeny of Tumor-Associated Macrophages

**DOI:** 10.3389/fimmu.2019.01799

**Published:** 2019-07-31

**Authors:** Marie Laviron, Alexandre Boissonnas

**Affiliations:** Sorbonne Université, INSERM, CNRS, Centre d'Immunologie et des Maladies Infectieuses - CIMI, Paris, France

**Keywords:** tumor-associated macrophage (TAM), cancer, ontogeny, monocyte-derived cell, cancer therapeutic

## Abstract

Tumor-associated macrophages (TAM) represent the main immune cell population of the tumor microenvironment in most cancer. For decades, TAM have been the focus of intense investigation to understand how they modulate the tumor microenvironment and their implication in therapy failure. One consensus is that TAM are considered to exclusively originate from circulating monocyte precursors released from the bone marrow, fitting the original dogma of tissue-resident macrophage ontogeny. A second consensus proposed that TAM harbor either a classically activated M1 or alternatively activated M2 polarization profile, with almost opposite anti- and pro-tumoral activity respectively. These fundamental pillars are now revised in face of the latest discoveries on macrophage biology. Embryonic-derived macrophages were recently characterized as major contributors to the pool of tissue-resident macrophages in many tissues. Their turnover with macrophages derived from precursors of adult hematopoiesis seems to follow a regulation at the subtissular level. This has shed light on an ever more complex macrophage diversity in the tumor microenvironment than once thought and raise the question of their respective implication in tumor development compared to classical monocyte-derived macrophages. These recent advances highlight that TAM have actually not fully revealed their usefulness and deserve to be reconsidered. Understanding the link between TAM ontogeny and their various functions in tumor growth and interaction with the immune system represents one of the future challenges for cancer therapy.

## Introduction

Tumor-associated macrophages (TAM) represent a major component of the tumor microenvironment (TME) that has been extensively studied in the past decades. They play a major role in tumor growth, metastatic dissemination, and therapy failure. Countless reports have described that TAMs can promote angiogenesis, inhibit the anti-tumor immune response, in particular T-cell-mediated cytotoxicity, support tumor growth, and secrete different factors involved in extracellular matrix (ECM) remodeling thus facilitating tumor cell motility and intravasation (1–6). High TAM infiltration is generally correlated with poor outcomes in several types of cancer, such as breast, ovarian, and lung cancer (7–9). However, in some indications TAM can be associated with enhanced anti-tumor immunity (10–12). Although macrophages were originally described as arising exclusively from circulating monocyte precursors (13), it was shown in the recent years that several organs harbor embryonic-derived populations of resident macrophages (ResMac) that maintain and self-renew throughout adulthood (14–16). This new concept challenges the dogma of TAM origin and questions their relative function. TAM subsets were originally classified as tumoricidal vs. tumor-promoting, often referred as M1/M2 macrophages (17), based on the expression of specific markers. However, the wide diversity of TAM cannot be covered by this nomenclature and many subsets express overlapping markers of the M1/M2 polarization (18–20). Whether TAM heterogeneity originates from their high plasticity or rather from independent specific lineages giving rise to multiple populations is still unclear. Although cellular ontogeny can recapitulate parts of the heterogeneity, it appears that environmental cues are also major determinants in cell education. Macrophage diversity would then be the result not only of ontogeny but also of niche-specific signaling events of tumor immunity (21–24). One can thus wonder whether the origin of TAM dictates their role in tumor development and is associated with various functions. This represent a key issue for anti-cancer therapies as these subsets might be differentially targeted regarding their role in tumor development.

## Macrophage Origin and Turnover

Although the precise origin of ResMac is still under debate [For the different models proposed, see review (14)], fate-mapping models highlighted a differential origin of tissue macrophages deriving either from an embryonic precursor (yolk sac, fetal liver) or a monocyte precursor from adult hematopoiesis origin. These precursors seed the tissues in different waves during development and adulthood giving rise to different ResMac. The dynamics of these waves vary between organs, age, and macrophage subsets. In some organs, such as the brain, the lung and the liver, some embryonic-derived ResMac (named here EmD-ResMac) maintain by self-renewal in adults whereas in the gut, the skin, the heart, and the pancreas most subsets are progressively replaced through the differentiation of monocyte precursors from adult hematopoiesis into monocyte-derived ResMac (named here MoD-ResMac) with different turnover rates. The ability of newly recruited macrophages to self-maintain in the tissue and become a ResMac *per se* is proposed to be tightly regulated by space availability and competition for growth factors in the niche (23).

This turnover appears to be variable among subsets in a given organ and could be induced by exposure to homeostatic environmental cues (e.g., mechanical, metabolic) specific of distinct subtissular regions. In the gut, long-lived macrophages with precise subtissular localization are key regulators of physiological functions (25). In the lungs, alveolar macrophages (AM) originate almost exclusively from yolk-sac derived macrophages and self-maintain throughout adulthood (26) whereas lung interstitial macrophages follow a more complex regulation, unveiling further heterogeneity in this subset (27, 28). While some of these interstitial macrophages have an embryonic origin (27), others differentiate from distinct monocyte precursors according to the subtissular niche they colonize, thus becoming the dominant population during adulthood (22). As most studies rest on relative proportion of the different subsets, whether EmD-ResMac are replaced or dominated by MoD-ResMac needs to be confirmed. Along tissue seeding, circulating monocytes undergo significant gene modifications to become truly ResMac sharing strong similarities with their counterpart of embryonic origin. This differentiation is dictated by lineage determining factors but mostly instructed by the local environment (29–31) as even mature macrophages adoptively transferred can be reprogrammed by the tissue to a certain extent (32, 33). Little information is available regarding the functional identity of MoD-ResMac and EmD-ResMac (34), but evidence show that macrophages derived from classical monocytes (named here MoD-Mac) infiltrating the tissue in an inflammatory context harbor distinct transcriptomic profiles, display shorter life span [reviewed in Guilliams et al. (35)] and can be functionally distinct (36).

## Reconsidering TAM Origin

The characterization of macrophage ontogeny in tissue at steady state has rapidly raised the question of their presence in neoplastic tissues and their differential role in tumor development.

Until recently, TAM were considered to originate exclusively from monocyte precursors undergoing differentiation upon tissue infiltration but the distinction of TAM from different origins led us to reconsider this dogma (37–39). In most cancer models, blocking the CCL2/CCR2 axis leads to a strong decrease in TAM abundance. Because CCR2 is a major receptor involved in monocyte trafficking, it has contributed to the idea that TAM originate from bone marrow-derived CCR2^+^ monocyte precursors (40–42). In an inducible lung carcinoma model, splenectomy resulted in a strong reduction in TAM. These spleen-derived TAM were shown to be also CCR2-dependent, suggesting that CCR2-deficiency does not necessarily account for a direct bone marrow provenance of TAM progenitors (43). However, deletion of *Ccr2* did not result in full depletion of macrophages suggesting that a CCR2-independent TAM accumulation or compensatory mechanisms might exist. CCR2-deficiency did not impact the relative proportion of TAM in the spontaneous PyMT-MMTV mammary carcinoma, but the use of *Ccr2*^*DTR*^ system led to an almost complete elimination of TAM suggesting their monocytic origin (44). However, CCR2 expression by ResTAM could not be excluded, and would also sensitize them to the toxin.

Recent studies have confirmed that TAM of different origins accumulate within the TME in mouse cancer models. Using parabiotic mice and bone marrow transfer, it was shown that the pool of TAM was composed of both newly recruited MoD-Mac and ResMac in a model of pancreatic ductal adenocarcinoma. Fate mapping models strongly support that a significant fraction of these ResTAM have embryonic origin and actively proliferate along with tumor growth (38). Although no difference in tumor weight was observed in *Ccr2*^−/−^ mice, ResTAM depletion using anti-CSF1R antibody and clodronate was associated with a strong reduction of tumor burden suggesting a dominant role of this population in tumor growth (38).

The expansion of resident interstitial macrophages with the development of multifocal lung tumors was also observed by Loyher et al. (37). Fate mapping experiments unveiled that at least a fraction of these TAM had an embryonic origin and greatly expand with tumor development. Interestingly AM, the typical embryonic-derived macrophages in the lung, did not expand and the relative proportion of ResTAM and recruited MoD-TAM was dependent on the anatomical niche of tumor development (37).

In the brain, conflicting results have been published regarding TAM origin (45). Microglial cells were shown to be the major contributor in several studies whereas others supported an accumulation of MoD-TAM (46–48). As several models used irradiation to test whether classical monocytes were able to replenish the brain, the disruption of the blood brain barrier may have artificially increased the accumulation of MoD-TAM (34, 49). Major contribution of this last population was demonstrated in primary and metastatic brain tumors (39). Different transcriptional profiles as well as different epigenetic landscapes were observed between microglia and MoD-TAM, associated with different activation patterns. The comparison with macrophages from healthy brain tissue revealed that some features shared by both TAM populations were not dependent on ontogeny but were “taught” by the TME. Additionally, CD49d was identified as a potent marker to discriminate microglia- vs. MoD-TAM in both murine models and human brain tumors (39).

Based on this work, single cell RNA sequencing was performed on macrophages from glioma or non-malignant human tissues (50). From 237 lineage-specific murine TAM genes, they compared the homologous genes in human samples and identified two TAM subsets that correlated with microglial enriched or bone marrow-derived TAM enriched genes. These two profiles were thus hypothesized to reflect the differential ontogeny of TAM in human brain tumors (50).

## Ontogeny as a New Feature of TAM Diversity

So far, few works support that TAM can be composed of newly recruited MoD-TAM mostly in a CCR2-dependent manner, but also ResTAM of embryonic origin (EmD-ResTAM) or arising from adult hematopoiesis (MoD-ResTAM) that locally proliferate and accumulate with tumor expansion. Whether this assumption can be generalized to other models deserve further investigation and the transposition to human tumors is even more hypothetical due to the lack of knowledge in macrophage ontogeny. Combining fate mapping models with RNA sequencing from mice to identify specific signature based on homologous human genes might be a valuable approach to track macrophage ontogeny in humans.

**Figure 1:**
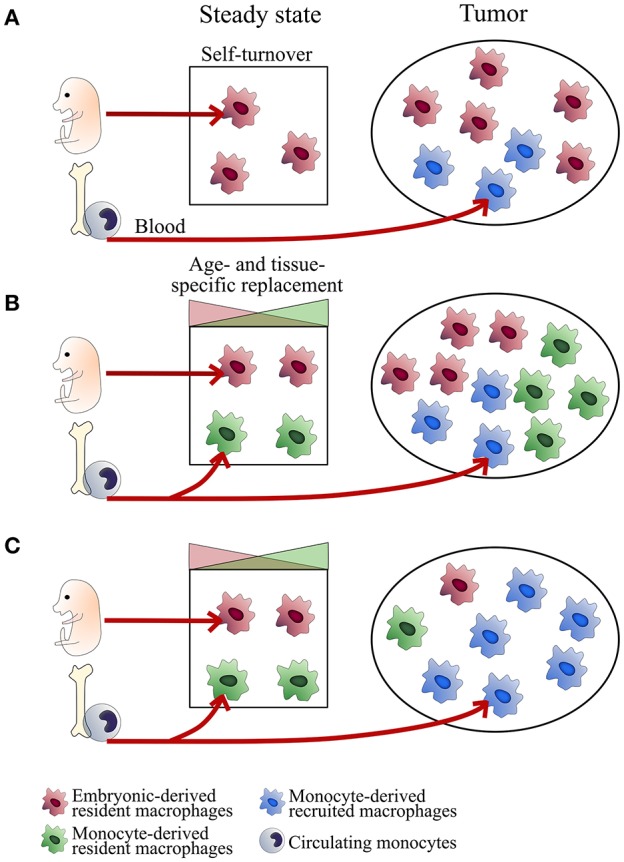
Tissue-dependent heterogeneity of TAM origin. Schemes represent different scenarios of TAM ontogeny. TAM composition may depend on the regulation of macrophage self-maintenance and turnover. This regulation is tissue- and subset-specific. In some tissues, embryonic-derived resident macrophages self-maintain over time (typically microglial cells in the brain, alveolar macrophages in the lungs; Scenario **A**). Other subsets are progressively replaced by monocyte-derived macrophages with turnover rates depending on the subtissular niches (typically, rapid turnover for certain macrophages of the gut or the dermis and slow turnover for interstitial macrophages of the lung, heart and pancreas; Scenario **B**). At tumor onset, classical monocytes are recruited to the tumor (mainly in a CCR2-dependent manner) and differentiate into inflammatory TAM (MoD-TAM). Depending on tumor localization and the inflammatory state, ResMac proliferate (scenarios **A** and **B**) or not (scenario **C**) and contribute more or less to the pool of TAM (ResTAM), exerting distinct functions in tumor development. Scenarios **(A,B)** are expected in brain and lung tumors respectively. The use of inflammatory ectopic tumor models may bias toward scenario **(C)**.

According to the model proposed for macrophages niches at steady state (23), the relative proportion of the different TAM subsets may vary with age, organs, subtissular niches, and the inflammatory state of tumor development (Figure 1). Understanding the relative importance of ResMac vs. MoD-Mac in the pool of TAM is limited by the lack of clear markers to discriminate them both in mice and human. Moreover, the use of experimental ectopic tumor models inducing local inflammation could bias the composition of the TAM compartment (scenario C in Figure 1). Ontogeny may represent a source of heterogeneity, hence an alternative classification in TAM diversity in addition to the common M1/M2 nomenclature.

## The M1/M2 Nomenclature Model in TAM Origin

The common characterization of TAM subsets relies on the M1/M2 polarization model induced by different *in vitro* stimuli (18). This model rapidly finds limitation in complex environments (*in vivo*) in which M1 and M2 stimuli can be present and generate very dynamic microanatomical niches. Tumors should be considered as an evolving tissue in which space availability and growth factors expression are changing over time (51, 52) and where inflammatory signals are generated by the loss of tissue integrity and immune cell infiltration (53). It is thus not surprising to find a wide range of activation profiles in the TME (18–20, 45). No typical M1/M2-associated marker defined one or the other TAM subset in lung unveiling heterogeneity among each subset (37). No direct link between TAM origin and the commonly described pro- or anti-tumor profile could be achieved in this study. One could expect that macrophage ontogeny and their anatomic localization define specific niches dictating their polarization toward a specific phenotype and function.

## TAM Function According to Their Origin

Despite recent works discriminating resident TAM vs. recruited TAM, their relative function in the TME has been barely addressed. The absence of phenotypic markers defining TAM according to their origin limits the possibility for functional studies. As previously mentioned, *Ccr2* deletion has been very useful to generate a TME with a largely reduced infiltration of recruited MoD-TAM while ResTAM seemed to be less affected. The variable extent of macrophage deletion observed between the different models may be related to the relative proportion of resident and recruited TAM. In most cases, the impaired macrophage accumulation in the TME was associated with a better control of the tumor and reduced metastatic dissemination (54–57) suggesting a major role for MoD-TAM in these processes. For instance, no difference in lung tumor burden was observed in CCR2-deficient mice compared to WT although nodules were smaller and more disperse suggesting that both MoD-TAM and ResTAM contributed to tumor growth but the presence of the former was associated with increased tumor cell spreading (37). Accordingly, CCL2 secretion by breast tumor cells activated Wnt-1 production by mammary intra-epithelial macrophages inducing an epithelial/mesenchymal transition-like signaling on cancer cells and driving early cancer dissemination (58).

In *Ccr2*^−/−^ mice engrafted with colorectal cancer, the reduction in TAM was associated with reduced tumor burden along with altered ECM composition (59). Genomic and proteomic analyses revealed upregulation of collagen synthesis and deposition in monocytes differentiating into TAM. CCR2-dependent TAM were shown to have a primary role in shaping the TME, thus promoting tumor expansion. On the other hand, Madsen and colleagues showed that CCR2^+^ MoD-TAM were responsible for collagen degradation in the TME in various tumor models. Transcriptomic analysis of these cells revealed a catabolic signature related to ECM degradation in this subset (60). These paradoxical observations suggest that different CCR2-dependent TAM subsets might be implicated in deposition and degradation of collagen in the TME. However, Res-TAM from pancreatic ductal adenocarcinoma were also shown to exhibit a pro-fibrotic profile, with increased expression of genes involved in ECM deposition and remodeling, which is a hallmark of this cancer. On the other hand, MoD-TAM were more efficient antigen-presenting cells (38).

Finally, in brain tumor, microglial-cells were enriched in pro-inflammatory genes as well as factors involved in ECM remodeling while MoD-TAM exhibited an immunosuppressive signature associated with immune suppression (39). In human glioma samples, MoD-TAM infiltration correlates with tumor grade. These TAM also exhibit an immunosuppressive profile with increased immunosuppressive cytokine expression. As observed by Chen et al. in a mouse model (61), these cells localize in necrotic regions and perivascular areas while microglia-derived TAM were found at the edge of the tumor (50).

Altogether, most studies rely on transcriptomic analysis and highlight functional profiles of resident vs. recruited TAM that cannot be fully associated with their origin across the different models. In addition, very little information is available regarding suppression of the adaptive response which is a key feature of TAM biology. Functional differences might be linked with the differential cues from the TME that polarize the macrophages in a niche-specific manner in addition to their ontogeny-specific features. Live imaging studies represent a complementary approach to compare functional difference between TAM subsets as reported in the lungs (37) and recently in the brain (62). Further studies using fluorescent strains and lineage-tracing models (63) will be necessary to better address the functional features of TAM subsets to better understand their role in tumor development as well as resistance to anti-cancer therapies and unveil key target for immunotherapy.

## Response of TAM Subsets to Anti-Cancer Therapies

Apart from their direct impact on tumor cells anti-cancer therapies display many immune-mediated effects. In addition to conventional treatments, many immunotherapies to boost the anti-tumor immune response are under investigation. TAM are usually considered as a factor of resistance to many therapies (64–66) but paradoxical roles in their efficacy are reported. Whether these contrasting roles are related to their ontogeny is unknown. Therefore, elucidating how TAM subsets are impacted by anti-cancer treatments is crucial especially in the context of combined therapies. So far, very few studies have addressed the selective targeting of TAM from different origins.

Following myeloablative chemotherapy using cyclophosphamide, we showed that both resident and recruited TAM were depleted by the alkylating agent in lung tumor (37). Recruited TAM rapidly recovered through a transient and massive wave of bone marrow-derived monocytes and TAM, while ResTAM recovery was much more limited. This wave contributed to tumor cell destruction and phagocytosis suggesting that in certain cases TAM are potent effector of the anti-tumor response. Specific targeting of TAM displaying protumor function without affecting tumoricidal activity is thus required in these conditions.

For instance, anti-CSF1R is quite efficient to deplete TAM in both human and mouse tumors (67) but its clinical efficacy is limited and leads to compensatory mechanisms (68). Mouse models suggest that anti-CSF1R treatment depletes efficiently certain subsets of ResMac but its effect on monocytes showed conflicting results that could be explained by variable dependency on CSF1R across different tumor microenvironment (45, 69, 70). In a lung tumor model, anti-CSF1R treatment blocked monocyte accumulation and differentiation into MHC-II^lo^ TAM, indicating a role for this axis in monocyte recruitment beyond CCL2/CCR2 (69). However, the impact on tumor growth was not reported. Another study in the lung showed strong depletion of TAM following anti-CSF1R administration although monocytes were not affected (70). No effect on tumor growth was observed, suggesting either that ResTAM are irrelevant to tumor growth or that some macrophage subsets involved in anti-tumor response could also be depleted. These studies were performed with different anti-CSF1R clones, which might have different pharmacological action.

PD1/PDL1 represents another promising approach to target macrophages as PD1 expression by macrophages increases along tumor growth (71). Anti-PD1 therapy was shown to induce a macrophage-dependent anti-tumor efficacy in a subcutaneous injected colon cancer cell line (71). Using bone marrow transplant of RFP^+^ cells it was shown that PD1^+^ TAM were mainly of medullar origin, although the use of fully reconstituted irradiated chimera may impact the compartment of resident MoD-TAM.

Restoring antigen presentation in the TME is essential to induce an effective T-cell anti-tumor response (72). The SIRPα/CD47 signaling axis is a “don't eat me” signal that is being hijacked by tumor cells to abrogate phagocytosis by TAM, thus impairing antigen processing. CD47 has been shown to be overexpressed in several cancer indications. Immunotherapy targeting CD47 has shown promising results in various tumors, including brain tumors (11, 73, 74). CD47 blockade was tested in glioblastoma pre-clinical models and showed a differential response of ResTAM vs. MoD-TAM. Both subsets showed enhanced phagocytosis upon treatment, but microglia-derived TAM displayed less inflammatory response. This was associated with prolonged mouse survival. The anti-CD47 effect on microglia was maintained in CCR2-deficient mice although the survival did not reach the same value as in WT mice (75). These results indicate that microglia-derived TAM might be the main subset involved in antigen presentation to T-cell in glioblastoma.

The development of immunotherapies targeting the myeloid compartment is challenging as targeting TAM is a double-edged sword process and the selective depletion of pro-tumoral macrophages without affecting the anti-tumor function would be idealistic.

## Concluding Remarks

The characterization of TAM ontogeny is still in its infancy. The lack of specific markers to discriminate and selectively target them for functional studies represents a technical limitation. Fate-mapping models and fluorescent reporters have revealed a differential contribution of tissue-resident and inflammatory macrophages in the pool of TAM in several tumor models, but no specific functional profile could be attributed to their origin across different cancer indication so far. Indeed, the contribution of TAM subsets follows complex spatio-temporal dynamics as macrophage niches evolves throughout life with specific regulation at the subtissular level depending on the organ and the age. Better characterization of how these subsets are differentially affected by anti-cancer therapy is of major importance to be able to selectively target them and thus promote the anti-tumor immune response.

## Author Contributions

ML and AB wrote the manuscript.

### Conflict of Interest Statement

The authors declare that the research was conducted in the absence of any commercial or financial relationships that could be construed as a potential conflict of interest.
